# Deficiency and Insufficiency of Vitamin D in Women of Childbearing Age: A Systematic Review and Meta-analysis

**DOI:** 10.1055/s-0042-1742409

**Published:** 2022-02-24

**Authors:** Rosa Camila Lucchetta, Isabele Held Lemos, Ana Luísa Rodriguez Gini, Sophia de Andrade Cavicchioli, Marcela Forgerini, Fabiana Rossi Varallo, Mariane Nunes de Nadai, Fernando Fernandez-Llimos, Patricia de Carvalho Mastroianni

**Affiliations:** 1 Department of Drugs and Medicines, School of Pharmaceutical Sciences, Universidade Estadual de São Paulo, São Paulo, SP, Brazil; 2 Universidade de Araraquara, Araraquara, São Paulo, SP, Brazil; 3 Department of Pharmaceutical Sciences, Faculty of Pharmaceutical Sciences of Ribeirão Preto, Universidade de São Paulo, Ribeirão Preto, SP, Brazil; 4 Department of Dentistry, Pediatric Dentistry and Public Health, Bauru School of Dentistry, Universidade de São Paulo, Bauru, SP, Brazil; 5 Department of Drug Sciences, Laboratory of Pharmacology, Faculty of Pharmacy, Universidade do Porto, Porto, Portugal

**Keywords:** cholecalciferol, vitamin D deficiency, nutritional epidemiology, maternal nutrition, women's health, colecalciferol, deficiência de vitamina D, epidemiologia nutricional, nutrição materna, saúde da mulher

## Abstract

**Objective** To estimate the prevalence of inadequate vitamin D level and its associated factors for women of childbearing age in Brazil.

**Methods** A systematic review was conducted (last updated May 2020). Meta-analyses were performed using the inverse-variance for fixed models with summary proportion calculation by Freeman-Tukey double arcsine. Reporting and methodological quality were assessed using the Joanna Briggs Institute tool for prevalence studies.

**Results** Our review identified 31 studies, comprising 4,006 participants. All the studies had at least one weakness, mainly due to the use of convenience sampling and small sample size. The overall prevalence of vitamin D deficiency, insufficiency, and both deficiency and insufficiency were 35% (confidence interval, 95%CI: 34–37%), 42% (95%CI: 41–44%), and 72% (95%CI: 71–74%), respectively.

**Conclusion** Although the magnitude of the prevalence of inadequate levels of vitamin D is uncertain, the evidence suggests that presence of vitamin D deficiency or insufficiency in women of reproductive age can cause moderate to severe problems.

## Resumo

**Objetivo** Estimar a prevalência de níveis inadequados de vitamina D e seus fatores associados para mulheres em idade fértil no Brasil.

**Métodos** Uma revisão sistemática foi realizada (última atualização em maio de 2020). As meta-análises foram realizadas usando o inverso da variância para o modelo fixo com cálculo de proporção sumarizada por transformação arco-seno duplo de Freeman-Tukey. A qualidade metodológica e de reporte foi avaliada usando a ferramenta do Joanna Briggs Institute para estudos de prevalência.

**Resultados** Nossa revisão identificou 31 estudos, compreendendo 4.006 participantes. Todos os estudos apresentaram pelo menos uma limitação, principalmente devido ao uso de amostra de conveniência e tamanho amostral pequeno. As prevalências gerais de deficiência, insuficiência e deficiência de vitamina D foram 35% (intervalo de confiança, IC 95%: 34–37%), 42% (IC 95%: 41–44%) e 72% (IC 95%: 71–74%), respectivamente.

**Conclusão** Embora a magnitude da prevalência de níveis inadequados de vitamina D seja incerta, a evidência sugere que presença de deficiência ou insuficiência de vitamina D em mulheres em idade reprodutiva pode causar problemas moderados a graves.

## Introduction

The deficiency and insufficiency of 25-hydroxyvitamin D, also known as 25(OH)D or vitamin D, is a worldwide issue: less than 50% of the world population has an adequate level of vitamin D, but in older people, pregnant women, and non-Western immigrants the proportion is smaller. 1 In pregnant women, for instance, the prevalence of insufficiency (25(OH)D < 50 nmol/L) and deficiency (25(OH)D < 25 nmol/L) ranged from 46% to 87% and 9% to 79%, respectively. 2 Even in warmer countries, such as Brazil, there is an alarming prevalence of vitamin D deficiency (28%) and insufficiency (45%), reaching 85% in pregnant women. 3 4

Recent studies suggested that vitamin D homeostasis may be important for several nonskeletal outcomes, including cardiovascular and respiratory diseases, neuromuscular function, psoriasis, falls, obesity, type 2 diabetes mellitus, multiple sclerosis, colorectal cancer, and coronavirus disease 19 (COVID-19). 5 6 7 8 9 10 11 12 Vitamin D deficiency also causes a series of poor gestational outcomes, 13 increasing the risk of preeclampsia and gestational diabetes mellitus, as well as the production of maternal inflammatory cytokines, 13 14 insulin resistance, 13 15 and postpartum depression. 13 16

In Brazil, there is a great variability in studies assessing insufficiency and deficiency of vitamin D in women of childbearing age (12–68%), 17 18 19 but there is also a lack of evidence that systematically summarizes their prevalence. A systematic review (2019) evaluated the deficiency and insufficiency of vitamin D in Brazil, with no specific analysis for women of childbearing age. 4 The present systematic review aimed to identify the prevalence and factors associated with inadequate levels of vitamin D in women of childbearing age in Brazil.

## Methods

### Study Design, Protocol, and Registration

A systematic review was performed in accordance with the Meta-analysis of Observational Studies in Epidemiology (MOOSE) group, 20 and Joanna Briggs Institute recommendations, 21 and reported following the Preferred Reporting Items for Systematic Reviews and Meta-Analyses (PRISMA). 22 The protocol of this review is available at Center for Open Science 23 and PROSPERO (CRD42020221605). This study is part of a larger project that evaluated vitamins A, B, C, D, and E, calcium, iodine, iron, and zinc deficiencies in women of childbearing age in Brazil.

### Information Sources, Search Strategy, and Eligibility Criteria

Electronic searches were conducted in the following databases: PubMed, Scopus, LILACS, World Health Organization (WHO), and CAPES' dissertations and theses (gray literature). The selection of these sources ensured including EMBASE, Medline, open access sources, scientific websites, and gray literature, 24 through a predefined search strategy (available in the protocol) 23 from their inception to May 2020. An additional manual search was performed using reference lists of reviews and included studies.

Studies that fulfilled the following criteria according to the CoCoPop acronym were included 25 : i) Condition: vitamin D deficiency or insufficiency; ii) Context: Brazil, without restriction of setting; iii) Population: women of childbearing age (15–49 years old) without any restriction of diseases or physiological status (e.g., nonpregnant, pregnant, postpartum). Data from studies that reported the deficiencies of interest, using a different population classification (e.g., premenopausal women), or different laboratory parameters were separated for appropriate subgroup analyses. All types of articles were included, except for reviews, letters, comments, case reports, and case series. No language restriction was applied.

### Study Selection and Data Extraction

Two researchers screened the titles and abstracts and evaluated the full-text articles independently. Discrepancies were solved in consensus meetings using another researcher as a referee.

Five researchers independently extracted the following data:

(i) Study characteristics (e.g., type of study, analysis period, state, region, funding, micronutrient assessed, and sampling method);
(ii) Participant characteristics (e.g., pregnant women, ethnicity, comorbidities, drug therapy or supplement in use, body mass index, age, education, per capita income);
(iii) Prevalence estimate, according to cutoff values used (n/N [%]) to total population and subgroups, when the information was available. When the studies reported vitamin D deficiency and insufficiency separately, we deduced the estimates considering the sum of participants.

### Synthesis of Results

Although predefined cutoffs for the assessment of deficiencies and insufficiencies of vitamin D were not considered inclusion criteria in the present review, only studies that considered identical cutoffs were grouped.

The data synthesis was primarily done by meta-analysis. Transitivity assessment was performed by comparing the CoCoPop acronym for each study. 25 Once important discrepancies were identified, sensitivity analyses with the exclusion of the study in question were performed (i.e., leave-one-out method). Proportion meta-analyses were conducted in the RStudio IDE (RStudio, PBC. Boston, MA, USA) software, version 3.6.3, 1.2.5033, 26 using the READR (RStudio, PBC.) 27 and META packages (RStudio, PBC.). 28

In the base-case, direct proportion meta-analyses were conducted using the inverse variance method. 28 To calculate the weighted summary proportion, the Freeman-Tukey double arcsine (PFT) was considered in the fixed effects model. 22 28 Although high heterogeneity is expected and, therefore, a random effects model could be considered appropriate, a fixed effects model is preferred for the assessment of prevalence, because otherwise the weighting will not properly consider the weight of the studies. 29 The result of the meta-analysis was given by the proportion combined with 95% confidence interval (95% CI), as well as the list of proportions (presented as a percentages), with their respective 95%CIs found in the individual studies. A Higgins inconsistency test (I ^2^ ) with an estimator for tau ^2^ was considered using the DerSimonian-Laird method.

Cumulative meta-analyses were also performed to assess changes and trends over time, and to highlight emerging or decreasing deficiency or insufficiency. Potential publication bias was assessed using rank tests with at least ten studies by meta-analysis. 28

Sensitivity analyses were performed by the leave-one-out method. Subgroup and meta-regression analyses, considering the publication year, state and region of Brazil, comorbidities, age, or status (i.e., not pregnant, pregnant, postpartum) were planned for meta-analyses with at least ten studies. Alternative statistical methods were also conducted to validate the conclusions (i.e., GLMM, Logit transformation, random effects, and Hartung and Knapp for random models).

### Methodological Quality in Individual Studies

An assessment of methodological and reporting quality based on the JBI Critical Appraisal Checklist for studies reporting prevalence data was conducted. 30 31 Two reviewers performed the assessment, independently. In the absence of consensus, points of disagreement were resolved by a third investigator.

### Data Sharing and Data Accessibility

The data that support the findings of this study are openly available in OSF at http:doi.org/10.17605/OSF.IO/J9QMH . 23

## Results

Our systematic review identified 1,977 records in the electronic databases after duplicate removal (PubMed, LILACS, and Scopus) and 91 additional records identified through other sources (manual search, WHO, and CAPES' dissertations and theses databases). After selection process, 31 studies were included, published between 2008 and 2020, reporting deficiency or insufficiency of vitamin D. The list of included and excluded studies, as well as a PRISMA flowchart, are available in the OSF. 23 Of the 31 studies selected, 23 were cross-sectional, 4 prospective, 2 retrospective cohorts, and 2 were case-control studies. The studies were conducted between 1995 and 2017 (six studies did not report inclusion period), in cities in the Southeast ( *n* = 18), South ( *n* = 7), Northeast ( *n* = 5), and Center-west ( *n* = 2) Brazilian regions, with women selected mainly from outpatient care ( *n* = 20). Araújo et al., 32 Queiroz, 33 Queiroz et al., 34 de Oliveira et al., 35 and dos Santos et al. 36 used a random probabilistic sampling, while Martins et al. 37 used convenience sampling ( Chart 1 ). 3 17 18 19 32 33 34 35 36 37 38 39 40 41 42 43 44 45 46 47 48 49 50 51 52 53 54 55 56 57 58 59 60 61 62 63 64 65 66 67 68

**Chart 1:** Description of the characteristics of the included studies

Study	Inclusion period	State/ region	Setting	Cutoff values	Funding
**Cross-sectional studies**					
Araújo et al. (2017), 32 Queiroz (2016), 33 and Queiroz et al. (2019) 34	Jun–Aug 2015	PB/NE	School	< 75 nmol/L	NR
Chrisostomo et al. (2018) 38	Jan–Mar or Jul–Aug 2016	PR/S	Obstetrical care	< 75 nmol/L 50–75 nmol/L < 50 nmol/L	NR
Duran de Campos et al. (2008) 39	Oct 1995–Jan 1999	SP/SE	Outpatient	25–50 nmol/L 12.5–25 nmol/L	NR
de Oliveira et al. (2020) 35	Feb 2013–Nov 2014	DF, RJ, RS, SC/S, SE, CW	School	< 75 nmol/L 50–75 nmol/L < 50 nmol/L	Brazilian Funding Authority for Studies and Projects, and CNPq
Souza et al. (2019) 40	Jan–Feb 2017	MA/NE	Outpatient	< 75 nmol/L 50–75 nmol/L < 50 nmol/L	NR
Delmonico et al. (2018) 41	2008–2016	RJ/SE	Outpatient	< 75 nmol/L	CAPES
Prado et al. (2015) 3	Dec 2011–Nov 2012	MG/SE	Obstetrical care	< 50 nmol/L	FAPEMIG
Ferreira et al. (2015) 42	NR	RJ/SE	Outpatient	< 50 nmol/L	FAPERJ
Flauzino et al. (2017) 43	Jul 2010–Mar 2011	PR/S	Outpatient	< 75 nmol/L	CAPES, CNPq, and UEL
Lopes et al. (2015) 44	2011–2013	SP/SE	Outpatient	< 75 nmol/L	FAPESP
Lopes et al. (2016) 45	Jan–May 2012	DF/CW	Outpatient	< 75 nmol/L 50–75 nmol/L < 50 nmol/L	NR
Machado et al. (2013) 46	May 2010–Dec 2011	SP/SE	University	< 75 nmol/L 50–80 nmol/L < 50 nmol/L	UNIFESP
Martins et al. (2018) 37	Oct–Dec 2016	CE/NE	Obstetrical care	< 75 nmol/L 50–75 nmol/L < 50 nmol/L	NR
Mendes et al. (2019) 47	NR	NR	NR	25–50 nmol/L	CNPq
Pena et al. (2015) 48	Nov 2012–Mar 2013	PE/NE	Obstetrical care	< 75 nmol/L 50–75 nmol/L < 50 nmol/L	CNPq
Pereira-Santos (2014) 49 and Pereira-Santos et al. (2018) 50	NR	BA/NE	Obstetrical care	< 75 nmol/L 50–75 nmol/L < 50 nmol/L	CNPq and CAPES
Peters et al. (2009) 51	Apr–May 2006	SP/SE	Outpatient/Rural	25–75 nmol/L	FAPESP
Santos et al. (2013) 52	Apr 2008–Sep 2010	PR/S	School	< 75 nmol/L 50–75 nmol/L < 50 nmol/L	CNPq
Santos et al. (2017) 53	NR	RS/S	Outpatient	< 75 nmol/L 50–75 nmol/L < 50 nmol/L	CNPq and CAPES
Santos et al. (2019) 54	2005–2012	RS/S	NR	< 50 nmol/L	CNPq, FAPERGS, and CAPES
Schtscherbyna et al. (2016) 55	Apr 2008–May 2011	RJ/SE	Outpatient	< 75 nmol/L	CAPES, FAPERJ, and CNPq
Shinjo et al. (2011) 56	NR	SP/SE	Outpatient	< 50 nmol/L	CNPq and Federico Foundation
Simões et al. (2016) 57	Apr 2013–Jun 2013	SP/SE	Obstetrical care	< 75 nmol/L 50–75 nmol/L < 50 nmol/L	FAPESP and CAPES
**Case-control**					
Dutra et al. (2019) 58	Sep 2016–Dec 2017	SP/SE	Obstetrical care	< 75 nmol/L 50–75 nmol/L < 50 nmol/L	CAPES, CNPq, and FAPESP
Menegati et al. (2016) 17	2006–2010	RJ/SE	Outpatient	< 75 nmol/L 50–75 nmol/L < 50 nmol/L	CAPES
**Prospective cohorts**					
Benaim et al. (2019) 59	Nov 2009–Oct 2011	RJ/SE	Outpatient	< 75 nmol/L 50–75 nmol/L < 50 nmol/L	CNPq and FAPERJ
Lepsch et al. (2017) 60 and Figueiredo et al. (2017, 2018, 2020) 61 62 63	Nov 2009–Oct 2011	RJ/SE	Obstetrical care	< 75 nmol/L 50–75 nmol/L < 50 nmol/L	CNPq and FAPERJ
Medeiros et al. (2016) 64	Mar 2010–Jul 2013	RJ/SE	Outpatient	< 75 nmol/L 50–75 nmol/L < 50 nmol/L	NR
Weinert et al. (2014, 2016) 65 66	Nov 2009–May 2012	RS/S	Obstetrical care	< 75 nmol/L 50–75 nmol/L < 50 nmol/L	Hospital de Clínicas de Porto Alegre
**Retrospective studies**					
Cruz et al. (2018, 2020) 18 19 67	Jan 2011–Jul 2015	RJ/SE	Outpatient	< 75 nmol/L 50–75 nmol/L < 50 nmol/L	FAPERJ
Rosa et al. (2013) 68	NR	RJ/SE	NR	38–225 nmol/L	NR

Abbreviations: NR, not reported; SD, standard deviation.

**State/Region** : BA, Bahia; CE, Ceará; CW, Center-west; DF, Distrito Federal; MA, Maranhão; MG, Minas Gerais; NE, Northeast; PB, Paraíba; PE, Pernambuco; PR, Paraná; RJ, Rio de Janeiro; RS, Rio Grande do Sul; S, South; SC, Santa Catarina; SE, Southeast; SP, São Paulo. **Funding/Institutions** : CAPES, Coordenação de Aperfeiçoamento de Pessoal de Nível Superior; CNPq, Conselho Nacional de Desenvolvimento Científico e Tecnológico; FAPEMIG, Fundação de Amparo à Pesquisa do Estado de Minas Gerais; FAPERGS, Fundação de Amparo à Pesquisa do Estado do Rio Grande do Sul; FAPERJ, Fundação de Amparo à Pesquisa do Estado do Rio de Janeiro; FAPESP, Fundação de Amparo à Pesquisa do Estado de São Paulo; UEL, Universidade Estadual de Londrina; UNIFESP, Universidade Federal de São Paulo.

Most studies assessed women of childbearing age ( *n* = 13), followed by pregnant women ( *n* = 10), adolescents ( *n* = 6), and postpartum women ( *n* = 4). Two studies assessed pregnant and nonpregnant women, concomitantly. Therefore, 4,006 participants were included, mainly women of childbearing age ( *n* = 1,239), with a mean age ranged from 13 to 46 years old, and mean body mass index ranged from 22 to 46 kg/m ^2^ . The majority of studies included women with a medical condition (e.g., HIV + , gestational diabetes mellitus, hypertension) or post Roux-en-Y gastric bypass surgery (RYGB, *n* = 18). Although drug therapy use was not reported is most studies, nutrient supplementation ( *n* = 11) or no supplementation ( *n* = 11) use were reported. The main characteristics of the participants are described in Chart 2 . 3 17 18 19 32 33 34 35 37 38 39 40 41 42 43 44 45 46 47 48 49 50 51 52 53 54 55 56 57 58 59 60 61 62 63 64 65 66

**Chart 2:** Description of the characteristics of the included participants

Study	Main characteristic (N)	Ethnicity	Comorbidities	Medicine/supplement	Body mass index, kg/m ^2^	Mean age, years
Araújo et al. (2017), 32 Queiroz (2016), 33 and Queiroz et al. (2019) 34	Adolescents (136)	Brown (62%)	NR (Excluded some conditions) ^a^	NR/None	Normal weight (72%)	17 (±SD 1) ^b^
Benaim et al. (2019) 59	Pregnant women (181)	Mixed (47%)	NR (Excluded some conditions) ^c^	NR/Yes	Median: 24 (IQR 22–27)	Median: 26 (IQR 22–31)
Chrisostomo et al. (2018) 38	Pregnant women (520)	Euro-descendant (52%)	Preeclampsia; GDM; HIV+	Antiretroviral/None	Median 31 (IQR: 27; 35)	Median: 30 (IQR: 25–35)
Cruz et al. (2018, 2020) 18 19 67	Pregnant and nonpregnant women (121)	NR	RYGB (Excluded some conditions) ^d^	NR/Yes ^e^	43 (±SD 3) to 44 (±SD 6)	30 (±SD 4) to 32 (±SD 4)
Rosa et al. (2013) 68	Women of childbearing age (56)	NR	RYGB	NR/Yes ^f^	46 (±SD 8)	35 (±SD 9)
Duran de Campos et al. (2008) 39	Women of childbearing age (30)	NR (excluded nonwhite)	RYGB	NR	29 (±SD 2.3) to 47 (±SD 8.6)	46 (±SD 3)
de Oliveira et al. (2020) 35	Adolescents (100)	Nonwhite (54%)	NR	NR	Normal weight (71%)	15–17 (59%)
Souza et al. (2019) 40	Pregnant women (71)	Dart (62%)	Healthy	NR/None	NR	26 (±SD 6)
Delmonico et al. (2018) 41	Women of childbearing age (20)	NR	Malignant breast lesions	NR	NR	37
Prado et al. (2015) 3	Postpartum women (226)	White (52%)	NR	NR/Yes (97%)	NR	28 (range 20–44)
Dutra et al. (2019) 58	Postpartum women (126) ^g^	NR	Hypertension (23%)	NR/Yes	26 (±SD 6) to 27 (±SD 5)	25 (±SD 7) to 26 (±SD 7)
Ferreira et al. (2015) 42	Women of childbearing age (73)	White (68%)	NR (Excluded some conditions) ^h^	NR/None	26 (±SD 1)	32 (±SD 1)
Flauzino et al. (2017) 43	Women of childbearing age (205)	Caucasian (71–78%) ^b^	HIV+	Antiretroviral/None	25 (±SD 0) to 26 (±SD 0) ^b^	40 (±SD 1) ^b^
Lepsch et al. (2017) 60 and Figueiredo et al. (2017, 2018, 2020) 61 62 63	Pregnant women (199)	Mixed (46%)	NR (Excluded some conditions)	None/None	< 25 (60%)	27 (±SD 6)
Lopes et al. (2015) 44	Adolescents (97)	NR	NR	NR	26 (±SD 9)	16 (±SD 1)
Lopes et al. (2016) 45	Women of childbearing age (369)	NR	Infertility and control	NR/None	NR	36 (±SD 4) to 37 (±SD 4)
Machado et al. (2013) 46	Pregnant women (49)	NR	HIV+	Antiretroviral/None	Excessive gestational weight (35%)	30 (±SD 7)
Martins et al. (2018) 37	Postpartum women (225)	Dark (79%)	Urinary tract infection (32%), hypertension (9%), GDM (1%), and bleeding (8%)	NR/Yes (64%)	Overweight or obesity (34%)	26 (±SD 7)
Medeiros et al. (2016) 64	Pregnant women (46)	NR	RYGB	NR/Yes ^i^	28 to 44 (±SD 6)	31 (±SD 5)
Mendes et al. (2019) 47	Women of childbearing age (79)	White (63%)	NR	NR/None	24 (±SD 5)	Median: 27 (IQR 24–31)
Menegati et al. (2016) 17	Women of childbearing age (58)	NR	RYGB and control (obesity) (Excluded some conditions) ^j^	NR/Yes (calcium)	35 (CI 95% 33–37) to 52 (CI 95% 40–73)	39 (CI 95% 36–42) to 40 (CI 95% 38–42)
Pena et al. (2015) 48	Pregnant and nonpregnant (179)	Nonwhite (82%)	Preeclampsia and gestational obesity	NR	IQR: 21–37	IQR: 19–33
Pereira-Santos (2014) 49 and Pereira-Santos et al. (2018) 50	Pregnant women (190)	Nonblack (68%)	NR (Excluded some conditions) ^k^	NR/Yes (5%)	Overweight (43%)	18–29 (63%)
Peters et al. (2009) 51	Adolescents (71)	NR (excluded nonwhite)	NR (Excluded some conditions) ^l^	NR	22 (±SD 0)	18 (±SD 1)
Santos et al. (2013) 52	Adolescents (198)	NR	Healthy	NR/None	Normal weight (76%)	13 (±SD 2)
Santos et al. (2017) 53	Women of childbearing age (102)	White (94%)	Polycystic ovary syndrome and controls	NR	27 (±SD 6) to 30 (±SD 6)	23 (±SD 7) to 25 (±SD 8)
Santos et al. (2019) 54	Women of childbearing age (61)	Caucasian (80%)	Healthy	NR/Yes (calcium and vitamin D)	29 (±SD 8)	37 (±SD 11)
Schtscherbyna et al. (2016) 55	Adolescents and young adults (35)	White (35%) ^b^	HIV+	Antiretroviral/NR	Normal (62%) ^b^	Around 18 (±SD 2) ^b^
Shinjo et al. (2011) 56	Women of childbearing age (20)	White (75%)	Juvenile onset of systemic sclerosis and controls	NR	NR	21 (±SD 2) to 21 (±SD 2)
Simões et al. (2016) 57	Postpartum women (99)	Blacks or mulatto (58%)	NR (Excluded some conditions) ^m^	NR/Yes (9%)	Overweight or obese (69%)	26 (±SD 5)
Weinert et al. (2014, 2016) 65 66	Pregnant women (184)	White (74%)	GDM (100%); Hypertension (22%)	NR/None	27 (±SD 5) to 30 (±SD 7)	32 (±SD 6)

Abbreviations: CI, confidence interval; GDM, gestational diabetes mellitus; HIV, human immunodeficiency virus; IQR, interquartile range; NR, not reported; RYGB, Roux-en-Y gastric bypass; SD, standard deviation. Notes: **A -** Pregnant, breastfed, carriers of chronic diseases (diabetes, hypertension, chronic kidney disease), chronic alcoholics, and chronic smokers were excluded. **B** - Both genders. **C -** Without any known infectious or chronic noncommunicable diseases (except obesity). **D -** Disabsorptive and restrictive surgeries prior to RYGB, disabsorptive syndromes, cancer and liver and/or kidney diseases (except hepatic steatosis), hypolipidemic or hypoglycemic use, active thyroid disorders, metabolic bone diseases, chronic use of diuretics or calcium channel blockers, female smokers, and presence of gestational diabetes were excluded. **E -** 850 mg of calcium carbonate and 600 IU of vitamin D3; when inadequacy of vitamin D was found in the preoperative period, all participants consumed 1500 IU of vitamin D; in addition, in case of pregnancy after RYGB, supplementation was adjusted from 1500 to 2000 IU vitamin D and 1200 mg after the immediate confirmation. **F -** Daily dietary supplementation of 500 mg of calcium carbonate and 400UI of vitamin D for an undetermined length of time. **G –** Mothers' full-term births, and mothers' preterm births. **H -** Women with smoking; eating disorders; major depression; any metabolic disease, such as diabetes mellitus or hypothyroidism; any chronic diseases severely affecting the CV, gastrointestinal, and renal systems; and pregnancy or lactation were excluded; **I -** 850 mg of calcium carbonate and 600 IU of vitamin D3. **J -** Women with malignant tumors or infectious diseases; were postmenopausal; were taking drugs that affect bone metabolism (bisphosphonates, estrogens, anticonvulsants, glucocorticoids); were pregnant; had malabsorption syndrome, primary hyperparathyroidism, renal, or liver failure; or weighed > 120 kg were excluded. **K -** Women with multiple pregnancies, preeclampsia, kidney problems, HIV and women who had not fasted for the blood collection were excluded. **L -** Chronic illness, pregnancy, and obesity were excluded. **M -** Alcohol use, hyperglycemia, hypertension, preterm/post-term deliveries and adolescent pregnancy, were excluded.

In the quality assessment, all studies had at least one ‘No’ answer, which suggests an overall poor reporting or methodological quality. The main questions with ‘No’ answers were regarding sample size ( *n* = 30) and sampling method ( *n* = 29). Questions with ‘Yes’ answers were about sample frame and valid methods used for the identification of the deficiencies. The detailed assessment of the methodological quality of the included studies is presented in Chart 3 . 3 17 18 19 32 33 34 35 37 38 39 40 41 42 43 44 45 46 47 48 49 50 51 52 53 54 55 56 57 58 59 60 61 62 63 64 65 66

**Chart 3:** Methodological and reporting quality assessment, considering the Joanna Briggs Institute tool for prevalence studies

	Question								
Study	1	2	3	4	5	6	7	8	9
Araújo et al. (2017), 32 Queiroz (2016), 33 and Queiroz et al. (2019) 34	Yes	Yes	No ^c^	Yes	Yes	N/A	Yes	Yes	No ^h^
Benaim et al. (2019) 59	Yes	No ^a^	No ^d^	No ^e^	Unclear	N/A	Yes	Yes	Unclear ^i^
Chrisostomo et al. (2018) 38	Yes	No ^a^	No ^d^	Yes	Yes	N/A	Yes	Yes	Yes ^j^
Cruz et al. (2018, 2020) 18 19 67	Yes	No ^a^	No ^d^	No ^e^	Unclear	N/A	Yes	No ^g^	No ^h^
Rosa et al. (2013) 68	Yes	No ^a^	No ^d^	No ^e^	Unclear	N/A	No ^f^	No ^g^	No ^h^
Duran de Campos et al. (2008) 39	Yes	No ^a^	No ^d^	No ^e^	Unclear	N/A	No ^f^	Yes	No ^h^
de Oliveira et al. (2020) 35	Yes	Yes	Yes	No ^e^	Unclear	N/A	Yes	Yes	No ^h^
Souza et al. (2019) 40	Yes	No ^a^	No ^d^	No ^e^	Unclear	N/A	Yes	Yes	No ^h^
Delmonico et al. (2018) 41	Yes	No ^a^	No ^c^	No ^e^	Unclear	N/A	Yes	Yes	No ^h^
Prado et al. (2015) 3	Yes	No ^a^	No ^d^	No ^e^	Unclear	N/A	Yes	Yes	Unclear ^i^
Dutra et al. (2019) 58	Yes	No ^a^	No ^d^	No ^e^	Unclear	N/A	Yes	No ^g^	No ^h^
Ferreira et al. (2015) 42	Yes	No ^a^	No ^d^	No ^e^	Unclear	N/A	Yes	Yes	No ^h^
Flauzino et al. (2017) 43	Yes	No ^a^	No ^d^	Yes	Yes	N/A	Yes	No ^g^	Unclear ^i^
Lepsch et al. (2017) 60 and Figueiredo et al. (2017, 2018, 2020) 61 62 63	Yes	No ^a^	No ^d^	Yes	Yes	N/A	Yes	No ^g^	Unclear ^i^
Lopes et al. (2015) 44	Yes	No ^a^	No ^d^	No ^e^	Unclear	N/A	Yes	Yes	No ^h^
Lopes et al. (2016) 45	Yes	No ^a^	No ^d^	No ^e^	Unclear	N/A	Yes	Yes	Yes ^j^
Machado et al. (2013) 46	Yes	No ^a^	No ^d^	Yes	Yes	N/A	Yes	Yes	No ^h^
Martins et al. (2018) 37	Yes	No ^b^	No ^d^	Yes	Yes	N/A	Yes	Yes	Unclear ^i^
Medeiros et al. (2016) 64	Yes	No ^a^	No ^d^	No ^e^	Unclear	N/A	Yes	Yes	No ^h^
Mendes et al. (2019) 47	Yes	No ^a^	No ^d^	No ^e^	Unclear	N/A	No ^f^	Yes	No ^h^
Menegati et al. (2016) 17	Yes	No ^a^	No ^d^	No ^e^	Unclear	N/A	Yes	Yes	No ^h^
Pena et al. (2015) 48	Yes	No ^a^	No ^d^	Yes	Yes	N/A	Yes	Yes	No ^h^
Pereira-Santos (2014) 49 and Pereira-Santos et al. (2018) 50	Yes	No ^a^	No ^d^	No ^e^	Unclear	N/A	Yes	Yes	Unclear ^i^
Peters et al. (2009) 51	Yes	No ^a^	No ^d^	No ^e^	Unclear	N/A	No ^f^	No ^g^	No ^h^
Santos et al. (2013) 52	Yes	No ^a^	No ^c^	No ^e^	Unclear	N/A	Yes	No ^g^	Unclear ^i^
Santos et al. (2017) 53	Yes	No ^a^	No ^d^	Yes	Yes	N/A	Yes	No ^g^	No ^h^
Santos et al. (2019) 54	Yes	No ^a^	No ^d^	Yes	Yes	N/A	Yes	Yes	No ^h^
Schtscherbyna et al. (2016) 55	Yes	No ^a^	No ^d^	Yes	Yes	N/A	Yes	Yes	No ^h^
Shinjo et al. (2011) 56	Yes	No ^a^	No ^d^	No ^e^	Unclear	N/A	Yes	Yes	No ^h^
Simões et al. (2016) 57	Yes	No ^a^	No ^d^	No ^e^	Unclear	N/A	Yes	Yes	No ^h^
Weinert et al. (2014, 2016) 65 66	Yes	No ^a^	No ^d^	Yes	Yes	N/A	Yes	Yes	Unclear ^i^

Abbreviation: N/A, not applicable. Notes: **1.** Was the sample frame appropriate to address the target population? **2.** Were study participants recruited in an appropriate way? **3.** Was the sample size adequate? **4.** Were the study's subjects and setting described in detail? **5.** Was data analysis conducted with sufficient coverage of the identified sample? **6.** Were valid methods used for the identification of the condition? **7.** Was the condition measured in a standard, reliable way for all participants? **8.** Was there appropriate statistical analysis? **9.** Was the response rate adequate, and if not, was the low response rate managed appropriately? **a** – Not reported, convenience sampling was considered. **b** – Reported convenience sampling. **c –** The target sample size reported was low. **d** – A target sample size was not reported. **e** – Did not report at least two of the following information: ethnicity, comorbidities, medicines/supplementation, body mass index, age, educational level, or income per capita. **f** – Vitamin D cutoff different than usual. **g –** Not reported numerator (n) or denominator (N) of prevalence). **h -** Not reported numerator (n) or denominator (N) of prevalence. **i** – The studies presented a response rate below 176 participants to vitamin D assessment. **j** – The studies presented a response rate between 176 and 345 (vitamin D), therefore is unclear if the sample size is appropriate, since a reliable estimate was not possible. **k** – The studies presented a response rate higher than 345, therefore, high confidence about good response was achieved.

Most studies ( *n* = 26) used common cutoff values (vitamin D deficiency: < 50 nmol/L or < 20 ng/mL; vitamin D insufficiency: 50–75 nmol/L or 20–30 ng/mL; and vitamin D deficiency or insufficiency: < 75 nmol/L or 30 ng/mL) ( Chart 1 ) and were included in meta-analyses.

**Fig. 1:**
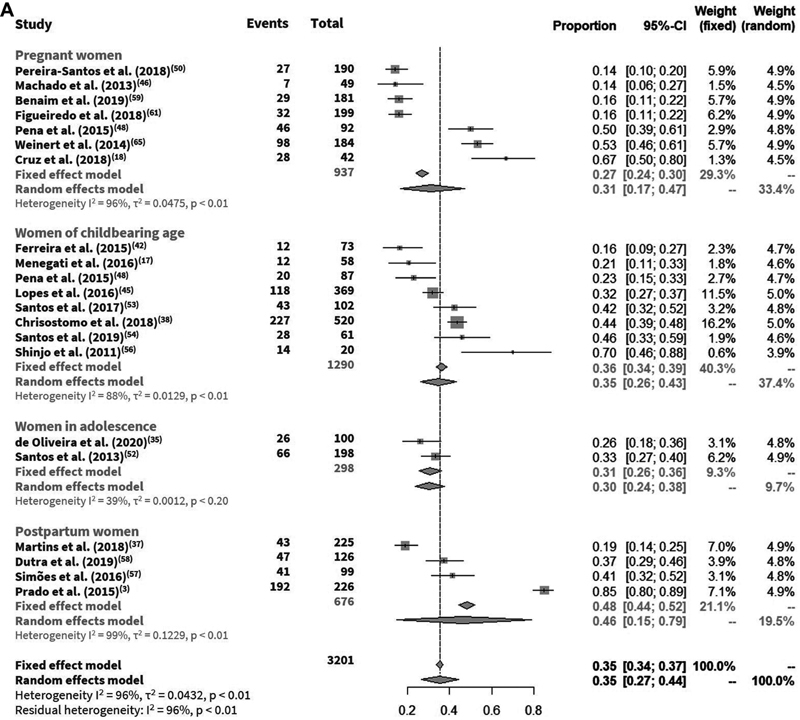
( **A** ) Vitamin D deficiency in pregnant women, women of childbearing age, women in adolescence, and postpartum women; ( **B** ) Vitamin D insufficiency in pregnant women, women of childbearing age, women in adolescence, and postpartum women.

The prevalence of vitamin D deficiency ranged from 3 to 85%, insufficiency from 15% to 68%, and deficiency or insufficiency from 34 to 94%. In the meta-analysis for the base-case, an overall prevalence of vitamin D deficiency of 35% (95%CI: 34–37%), insufficiency of 42% (95%CI: 41–44%) ( Fig. 1 ), and deficiency or insufficiency of 72% (95%CI: 71–74%) ( Appendix A, supplementary material ) were obtained. 23 When the population subgroups were considered, lower and higher prevalence of vitamin D deficiency were identified in pregnant (27%) and postpartum women (48%), respectively; and lower and higher prevalence of vitamin D insufficiency were associated with adolescents (37%) and women of childbearing age (50%) ( Fig. 1 ).

**Figure:**
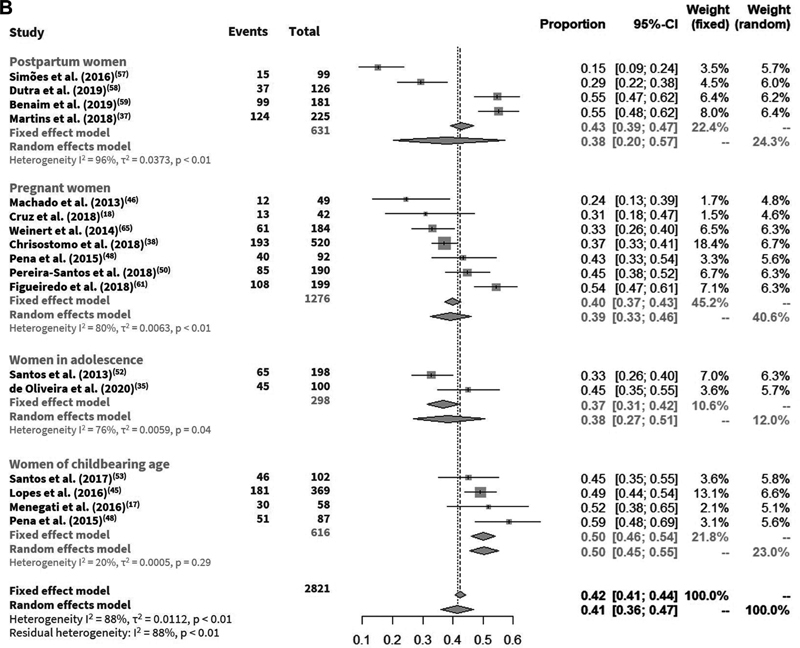


**Fig. 2:**
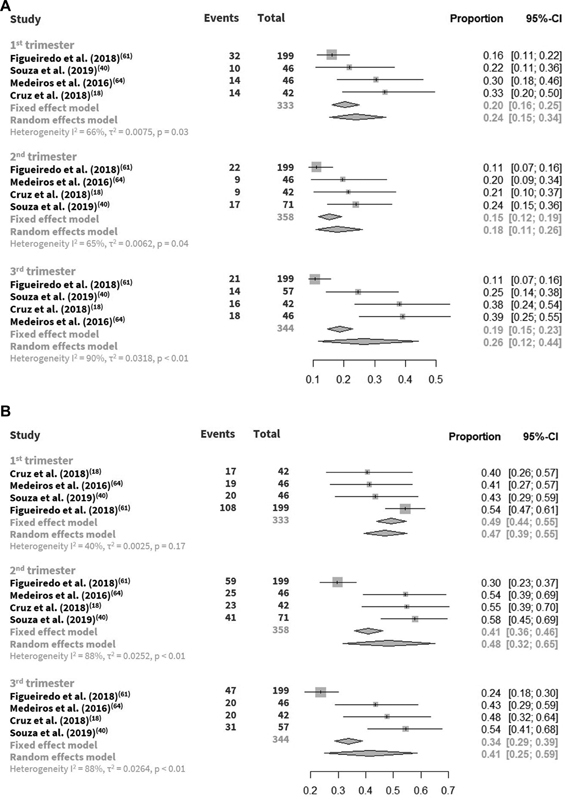
( **A** ) Vitamin D deficiency along gestational trimesters; ( **B** ) Vitamin D insufficiency along gestational trimesters.

Some studies reported subgroup analyses: higher deficiency or insufficiency prevalence values were found in adolescence ( *p* -value = 0.02), 40 first pregnancy ( *p* = 0.01), 40 ≥ 11 years of schooling ( *p* = 0.03), 49 50 first gestational trimester ( *p* = 0.01), 49 50 face and hands exposed to the sun ( *p* = 0.01), 49 50 methods of commuting by motor vehicles ( *p* = 0.01), 49 50 and winter ( *p* < 0.001). 49 50 60 61 62 63 Except for gestational trimester, no meta-analyses for these subgroups were possible due to the small number of studies in each subgroup, or different categorization for the same subgroup. Four studies assessed vitamin D status throughout gestational trimesters, with little variation among trimesters of vitamin D deficiency (15–20%) or insufficiency (34–49%) and wide confidence intervals ( Fig. 2 ).

Five studies assessed vitamin D in women post-RYGB, and two of them analyzed pregnant women after RYGB. No meta-analysis was possible due to the different cutoff values and categories used. The deficiency, insufficiency, and deficiency or insufficiency ranged from 12 to 39%, 41 to 54%, and 60 to 91%, respectively.

Cumulative meta-analyses were performed considering the year of publication, showing a trend toward a lower prevalence of vitamin D deficiency, and higher prevalence of vitamin D insufficiency and vitamin D deficiency or insufficiency, with a slight join point in 2017 ( Appendix B, supplementary material ). 23 Meta-regression analyses were conducted for publication year, and a moderator effect was not identified ( *p* > 0.05) ( Appendix C, supplementary material ). 23 Meta-regression or subgroup analyses for other variables were not possible, and neither were cumulative meta-analyses regarding gestational trimesters, because the minimum number of studies required was not met.

Sensitivity analyses by the leave-one-out method were not able to reduce heterogeneity (93–96%) and the overall prevalence ranged from 32 to 37% for vitamin D deficiency, 41 to 44% for vitamin D insufficiency, and 71 to 73% for vitamin D deficiency or insufficiency ( Appendix D, supplementary material ). 23 The study with more influence in the variations was Prado et al., 3 conducted in Minas Gerais, in 2012, with postpartum women taking supplements (97%). Sensitivity analyses with alternative statistical methods identified values of prevalence ranging from 35 to 37% for vitamin D deficiency, 41 to 43% for vitamin D insufficiency, and 69 to 72% for vitamin D deficiency or insufficiency ( Appendix A, supplementary material ). 23 It was not possible to conduct sensitivity analyses regarding gestational trimesters.

Potential publication biases were not identified in vitamin D deficiency ( *p* = 0.84), insufficiency ( *p* = 0.60), or deficiency or insufficiency ( *p* = 0.54) in statistical or visual analyses ( Appendix E, supplementary material ). 23 It was also not possible to conduct statistical and visual analyses of publication bias for meta-analysis along gestational trimesters.

Four studies reported different cutoff values and were not included in any meta-analysis. They identified prevalence values ranging from 11% to 75%: Duran de Campos et al. 39 identified serum 25(OH)D levels between 12.5 and 25 nmol/L (5–10 ng/mL) in 50% of the participants, and between 25 and 50 nmol/L (10–20 ng/mL) in 40% of the participants; Mendes et al. 47 identified 11% of the participants with values between 25 and 50 nmol/L; Peters et al. 51 identified 61% of the participants with values between 25 and 75 nmol/L (11–30 ng/mL); and Rosa et al. 68 identified 55% and 75% of the participants with values between 15 and 90 ng/mL in pre- and postoperative RYGB, respectively.

## Discussion

In this systematic review, 31 studies assessing prevalence of inadequate levels of vitamin D in women of childbearing age were found, reporting vitamin D deficiency (3–85%), insufficiency (15–68%), and deficiency or insufficiency (34–94%), with a mean prevalence of 35%, 42%, and 72% identified through the meta-analysis, respectively.

Redundant evidence of vitamin D levels was identified, especially for women of childbearing age in Brazil, to the detriment of population subgroups such as pregnant women, women who have recently given birth, and adolescents. In 2019, Pereira-Santos et al. 4 identified 72 studies that reported prevalence of vitamin D deficiency (28%) and insufficiency (45%) in the general population, and five studies that reported prevalence of 33% and 49%, respectively, in pregnant women. Although our systematic review identified the double of studies in pregnant women and 22 studies with women of childbearing age, our prevalence is similar to the Pereira-Santos' et al. 4 study, confirming the findings of our cumulative meta-analysis that new studies (published after 2017) had little impact on the prevalence estimates. At the same time, all the included studies showed weaknesses and high heterogeneity, which reduced the confidence on the prevalence rates reported.

Although little variation on the estimates has been added in the last years for women of childbearing age, when considering population subgroups (e.g., adolescents, pregnant women, postpartum women) the uncertainty still exists. For instance, when considered vitamin D deficiency in postpartum women (48%, 95% CI 44–52%, I ^2^ 99%), Martins et al. 37 identified prevalence of 19%, whereas Prado et al. 3 described it as 85%. While Martins et al. 37 included 79% of dark-skin women (variable associated with deficiency), 64% using supplement (variable associated with sufficiency), and during spring and summer (variable associated with sufficiency); Prado et al. 3 included 52% of white women (variable associated with sufficiency), 97% using supplement (variable associated with sufficiency), and throughout the year.

Moreover, it was not possible to conduct a robust subgroup analysis to explore the heterogeneity, as well as to identify possible associated factors to deficiency or insufficiency of vitamin D, since most studies did not report the characteristics of the participants, nor population subgroup analysis using common categories. Primary studies should appropriately report the findings according to common subgroups, and minimally, season, skin pigmentation, WHO standardized age group, 69 and supplement use.

In comparison with international data for inadequate vitamin D levels, our prevalence estimates are lower than estimates for women in Iran (44% deficiency), 70 and for women of childbearing age in in Saudi Arabia (77% deficiency or insufficiency), 71 but higher than estimates for adolescent girls in India (26% deficiency). 72 Several factors can explain the differences between the estimates, such as age, latitude, skin pigmentation, dietary habits, fortification of foods with vitamin D, use of vitamin D supplements, sunlight exposure, and cultural factors. 1 73 74 75 To exemplify, Gomes et al. 76 identified a seriously inadequate intake of vitamin D among Brazilian pregnant women in the primary healthcare network.

It is important to highlight that our systematic review identified several studies evaluating nonpregnant and nonlactating women, which were grouped as women of childbearing age. Notwithstanding, it was noted that many of these women had conditions associated with inadequate levels of vitamin D, such as overweight or obesity, 77 78 79 gestational diabetes mellitus, 80 preeclampsia, 81 82 cardiovascular disease, 83 breast cancer, 84 polycystic ovarian syndrome, 85 and infertility, 86 among others, which may overestimate the identified prevalence.

Another important consideration is that despite the variation in cutoff values used by studies to define vitamin D deficiency, most studies included in this meta-analysis considered the threshold recommended by the US Institute of Medicine (< 50 nmol/L of 25 (OH)D) as opposed to the threshold recommended by the Endocrine Society Practice Guidelines (< 75 nmol/L of 25(OH)D). The generally accepted cutoff levels consider the values necessary to ensure optimal effects in the calcium economy and skeletal health, 87 and studies designed to assess the correlation of clinical responses with clinically relevant vitamin D deficiency suggest that depending on the physiological parameters considered (e.g., pregnancy outcomes, cardiometabolic risk) the results may differ and be even greater than those mentioned above, 88 89 90 resulting in the identification of larger populations with vitamin D deficiency. Although it is not possible to be sure about the magnitude of deficiency/insufficiency of vitamin D in some subgroups among Brazilian women, current evidence suggests that this is a public health problem, given the Institute of Medicine's (US) recommended cutoff values. 91 In this sense, some preventive strategies for adequate vitamin D levels include fish consumption, food fortification, 92 and advice on moderate sunlight exposure. 1 93

Among the few countries with specific policies, the United Kingdom and Finland stand out with the recommendation of 10 μg of vitamin D daily intake for general population, and the mandatory food fortification programs, respectively. 94 In pregnant women, conflicting evidence suggests the benefit of supplementation, despite the documented negative clinical, humanistic, and economic impact of the deficiency or insufficiency of vitamin D, mainly, during the first trimester of pregnancy. 95 The hesitation about the recommendation of supplement intake may be justified by the limited evidence on the safety of vitamin D supplements, which could explain the reason why WHO does not recommend the supplementation during pregnancy as part of routine antenatal care. 96 Conversely to WHO, the Brazilian consensus recommends supplementation in pregnant women at risk of deficiency. 11 However, the Brazilian consensus does not recommend generalized vitamin D supplementation for the entire population, while it recommends the assessment of serum levels in obese patients. 11

Despite several options of vitamin supplements containing vitamin D being available in Brazil, with some of them included in Brazilian National List of Essential Medicines (Rename), 97 no national policy to prevent vitamin D insufficiency or deficiency in any women subgroup exists. In addition to funding studies to estimate the prevalence of micronutrient deficiencies in women of childbearing age, 98 a government policy is needed to avoid vitamin D inadequate levels, as well as excessive intake by self-medication or inappropriate prescription. 99

As any systematic review, one limitation of this study is that missing studies could exist. To overcome this limitation, extensive gray literature and manual searches to find unpublished and published studies were conducted, having found a few studies not retrieved by electronic searches. Although a high number of studies were identified through manual search, which could be seen as a limitation of the search strategy, one hypothesis is that many studies may not have properly written titles and abstracts, or are not correctly indexed, hindering the automatic search algorithm's ability to retrieve them. Finally, another limitation was the absence of a robust analysis about potential associated factors of inadequate levels of vitamin D, due to the poor reporting of the compiled studies.

## Conclusion

Although the magnitude of the prevalence of inadequate levels of vitamin D is uncertain, the evidence found in the literature suggests a moderate to severe problem with a prevalence of vitamin D deficiency (35%), insufficiency (42%), and deficiency or insufficiency (72%) in women of reproductive age. Future studies about vitamin D levels should consider random probabilistic sampling, appropriate sample sizes and reporting of findings. Furthermore, vitamin D studies should consider estimates according to the season, skin pigmentation, age range standardized by WHO, and use of supplements, to better inform potential health policies.
