# Supplementary material for: Deficiency and Insufficiency of Vitamin D in Women of Childbearing Age: A Systematic Review and Meta-analysis
Source: Rev Bras Ginecol Obstet. 2022 Feb 24;44(4):409–24. doi: 10.1055/s-0042-1742409 (PMC9948108; doi:10.1055/s-0042-1742409)
Supplement: Supplementary file 1 — Supplementary Material [file 10-1055-s-0042-1742409-s210142.pdf]

Appendix A Sensitivity Analysis – Alternative statistical method

Alternative Statistical Method	Prevalence (CI 95%), I^2		
**Base case**	**Vitamin D deficiency among population subgroups**	**Vitamin D insufficiency among population subgroups**	**Vitamin D deficiency or insufficiency among population subgroups**
- Inverse variance method<br>- Fixed model<br>- DerSimonian-Laird estimator for tau^2<br>- Jackson method for confidence interval of tau^2 and tau<br>- Freeman-Tukey double arc transformation<br>- Clopper-Pearson confidence interval for individual studies	0.35 [0.34; 0.37], 96.3%	0.42 [0.41; 0.44], 87.8%	0.72 [0.71; 0.74], 91.0%
**Sensitivity Analysis**			
- Inverse variance method<br>- Random model<br>- DerSimonian-Laird estimator for tau^2<br>- Jackson method for confidence interval of tau^2 and tau<br>- Freeman-Tukey double arc transformation<br>- Clopper-Pearson confidence interval for individual studies	0.35 [0.27; 0.44], 96.3%	0.41 [0.36; 0.47], 87.8%	0.71 [0.65; 0.77], 91.0%
- Inverse variance method<br>- Fixed model<br>- DerSimonian-Laird estimator for tau^2<br>- Jackson method for confidence interval of tau^2 and tau<br>- Logit Transformation<br>- Clopper-Pearson confidence interval for individual studies	0.37 [0.35; 0.38], 94.8%	0.43 [0.41; 0.45], 86.3%	0.69 [0.68; 0.71], 88.2%
- Random intercept logistic regression model<br>- Fixed model<br>- Maximum likelihood estimator for tau ^ 2<br>- Logit Transformation<br>- Clopper-Pearson confidence interval for individual studies	0.36 [0.34; 0.38], 95.9%	0.43 [0.41; 0.45], 89.4%	0.71 [0.70; 0.73], 94.2%
- Inverse variance method<br>- Random model<br>- DerSimonian-Laird estimator for tau^2<br>- Jackson method for confidence interval of tau^2 and tau<br>- Adjustment of Hartung-Knapp model for random effects<br>- Freeman-Tukey double arc transformation<br>- Clopper-Pearson confidence interval for individual studies	0.35 [0.34; 0.37], 96.3%	0.41 [0.35; 0.48], 87.8%	0.71 [0.63; 0.79], 91.0%

Note: Underlining was used to highlight the difference of the method from the base case.

 © 2022. Federação Brasileira de Ginecologia e Obstetrícia. All rights reserved.

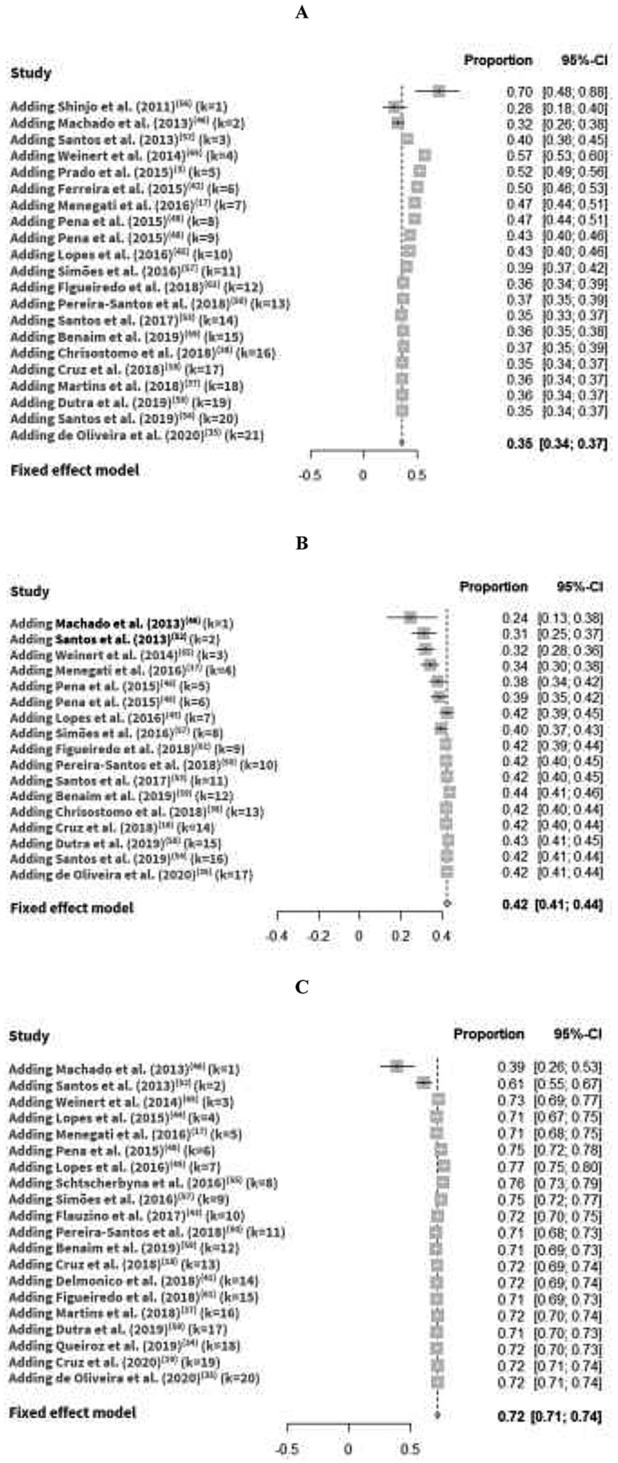

Appendix B Cumulative analysis: Year – a) vitamin D Deficiency, b) Vitamin D Insufficiency, and c) Vitamin D Deficiency or Insufficiency among population subgroups.

 © 2022. Federação Brasileira de Ginecologia e Obstetrícia. All rights reserved.

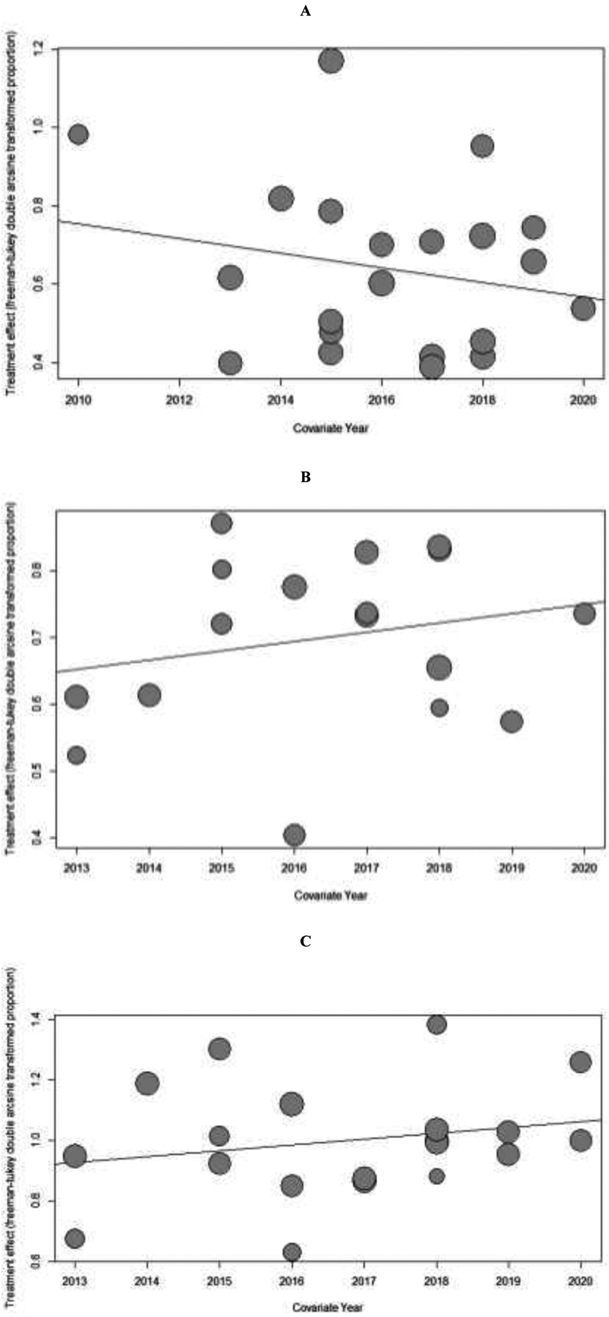

Appendix C Meta-regression analyses: Year – A) Vitamin D Deficiency, B) Vitamin D Insufficiency, and C) Vitamin D Deficiency or Insufficiency among population subgroups.

 © 2022. Federação Brasileira de Ginecologia e Obstetrícia. All rights reserved.

A

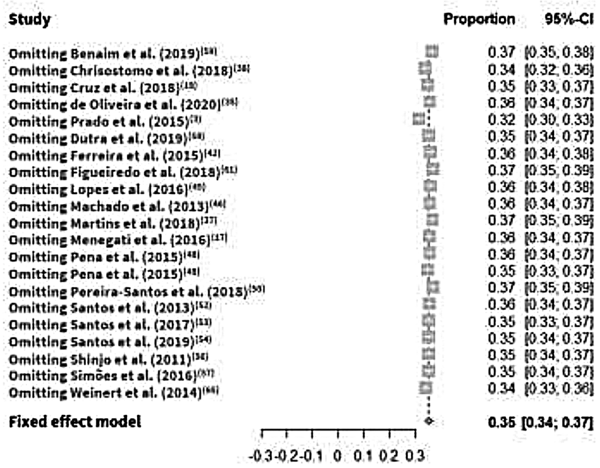

B

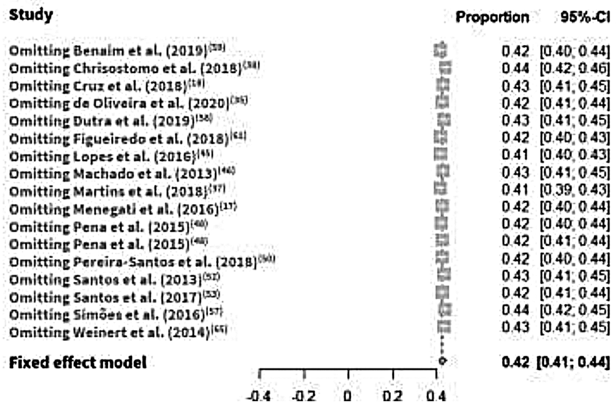

C

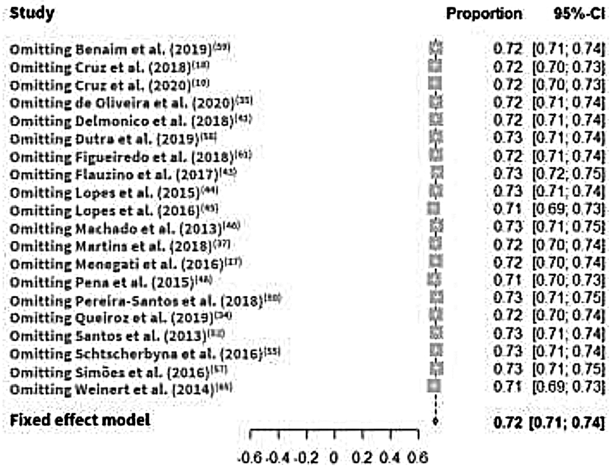

Appendix D Sensitivity analysis, leave-one-out method: a) Vitamin D deficiency, b) Vitamin D Insufficiency, and c) Vitamin D Deficiency or Insufficiency among population subgroups.

 © 2022. Federação Brasileira de Ginecologia e Obstetrícia. All rights reserved.

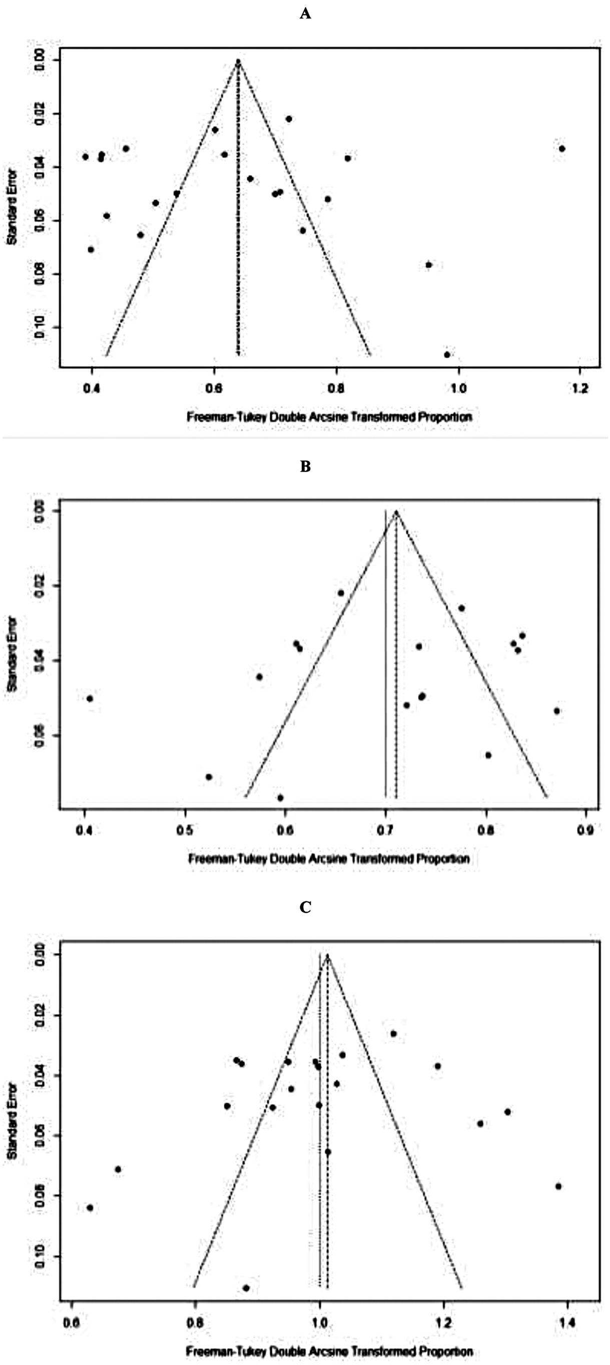

Appendix E Publication bias potential analysis – funnel graphs: a) Vitamin D Deficiency ($p = 0.84$), b) Vitamin D Insufficiency ($p = 0.60$), c) Vitamin D Deficiency or Insufficiency ($p = 0.54$) among population subgroups.

 © 2022. Federação Brasileira de Ginecologia e Obstetrícia. All rights reserved.
